# Myocardial T1 mapping using SMART1Map: initial *in vivo* experience

**DOI:** 10.1186/1532-429X-15-S1-P13

**Published:** 2013-01-30

**Authors:** Jeff A Stainsby, Glenn S Slavin

**Affiliations:** 1 GE Healthcare, Toronto, ON, Canada; 2 GE Healthcare, Bethesda, MD, USA

## Background

Recently a single-point, saturation-recovery myocardial T1 mapping sequence (SMART1Map = Saturation Method using Adaptive Recovery Times for cardiac T1 Mapping) was presented [1]. Compared to common methods like MOLLI, SMART1Map measures true T1 instead of apparent T1 relaxation, is more time efficient and can track exact TI times instead of estimated ones based on the heart rate during prescription. In this work we compare SMART1Map to MOLLI in an initial cohort of volunteers.

## Methods

Versions of SMART1Map and MOLLI were implemented on a GE MR450w scanner. Four healthy subjects were imaged without contrast agent and with informed consent. Single shot images were acquired at the TI times. SMART1Map acquisitions collected 5 TIs in 9 heart beats at 1,1,1,2 and 4 heart beats after saturation. MOLLI acquisitions collected 8 TIs (2,2 and 4 heartbeats after each inversion) in 14 heart beats. A total of 12 data sets for each sequence were collected. T1 values in an ROI in the mid-septum were calculated. Values were computed with both estimated TI times based on the heart rate at the time of scan prescription and true TI times based on pulse sequence measurements of each cardiac interval during scanning. MOLLI values were computed with and without Look-Locker (LL) correction [2]. SMART1Map values represent true, not apparent, T1 values and thus no correction was applied.

## Results

**Figure 1:**
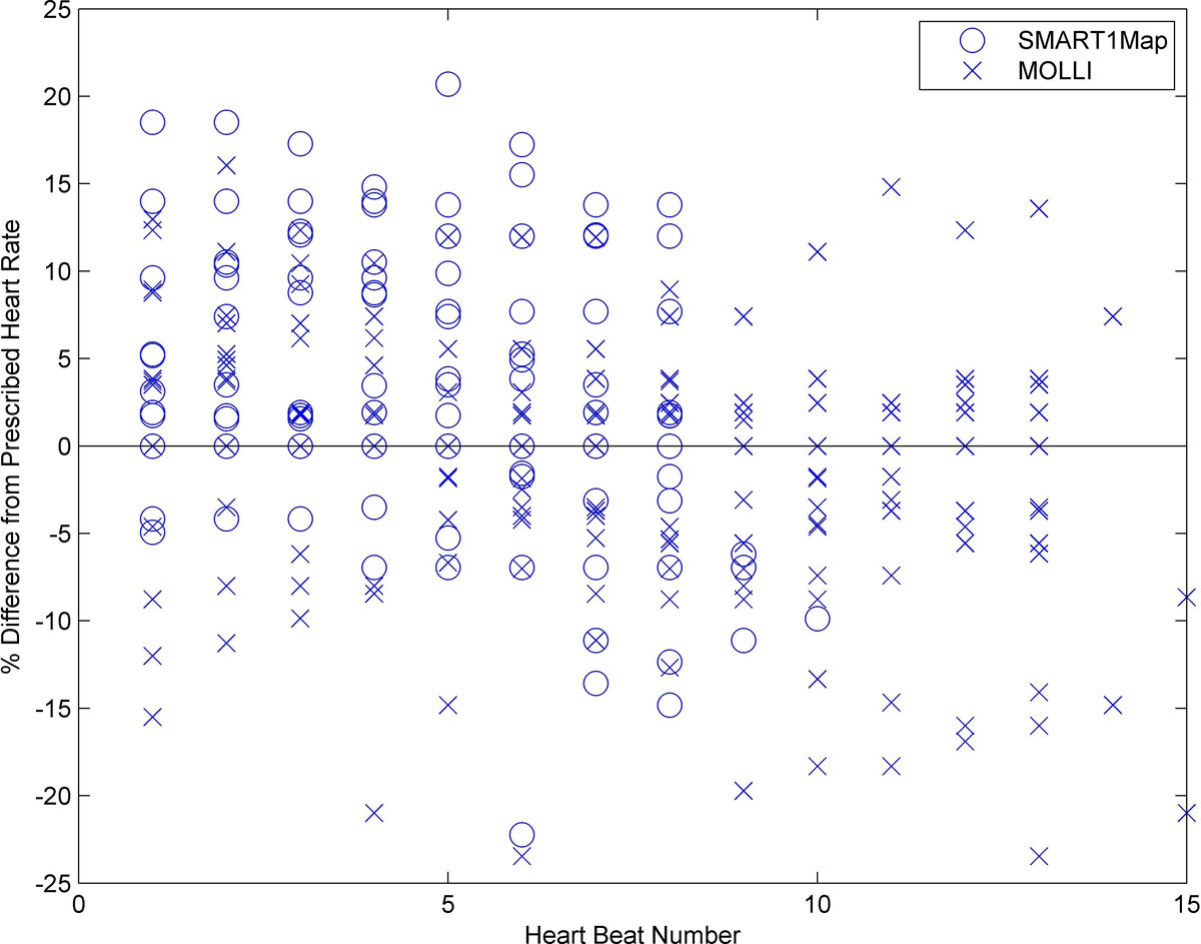
For all acquisitions the pulse sequence recorded in real-time the actual duration of each RR interval during scanning from which an instantaneous heart rate is determined. The variation in this beat-by-beat heart rate as a percentage of the fixed heart rate determined at the scan prescription stage is plotted as a function of heart beat number during the scan. Data for SMART1Map (o's) and MOLLI (x's) are shown and demonstrate heart rate variations can reach 20% across even these short breath-held scans.

Measured T1 values are summarized in Table 1. The mean T1 from SMART1Map (1193 ms) is consistent with T1 values reported in the literature using single-point methods: T1=1219 ms, [3] and T1=1175 ms [4]. SMART1Map acquires its two long (>1RR) TI samples in two separate blocks of multiple (2 and 4) heartbeats so it is important to account for the effect of heart rate variations on the actual TI times to achieve robust T1 measurements. This is demonstrated by the difference in T1s when using estimated and actual TIs for SMART1Map as in Table 1. Figure 1 plots heart rate variability over all series in these subjects relative to the fixed rate at scan prescription, illustrating 20% variations in heart rate over a breath hold were seen. MOLLI demonstrates less benefit from the use of actual TI times, resulting in T1 values that remain underestimated relative to the expected T1s. This may be due to a variety of confounding factors including the interleaving of TIs, the influence of differential signal recovery, and the behavior of the LL correction, which is not clearly understood and may obscure the effects of heart rate variation.

**Table 1:** Mean measured T1 values in normal myocardium.

Mean T1 values (N=12)	T1 (ms)
MOLLI no LL correction	792.1
MOLLI with LL correction and estimated TI times	1018.2
MOLLI with LL correction and measured TI times	1012.8
SMART1Map with estimated TI times	1279.1
SMART1Map with measured (true) TI times	1193.0

Three series for each sequence in each of 4 volunteers were acquired. Values measured using a version of MOLLI with no correction, with the common Look-Locker correction and with the Look-Locker correction and correcting TI times based on pulse sequence-measured RR durations are listed. Values measured using SMART1Map with estimated TI times and the corrected TI times are also listed. The T1 values from SMART1Map with measured TI times closely match unenhanced myocardium T1's measured with single-point methods in the literature.

## Conclusions

SMART1Map is a new T1 mapping method that provides accurate, breath held, true myocardial T1 values for normal myocardium. Compared to literature values, it provides more accurate T1 measurements compared to MOLLI which studies have shown underestimates long T1s.

## Funding

N/A
